# Comparative Study for the Determination of Fat-Soluble Vitamins in Rice Cereal Baby Foods Using HPLC-DAD and UHPLC-APCI-MS/MS

**DOI:** 10.3390/foods10030648

**Published:** 2021-03-18

**Authors:** Maria Katsa, Natalia Papalouka, Theodora Mavrogianni, Irene Papagiannopoulou, Marios Kostakis, Charalampos Proestos, Nikolaos S. Thomaidis

**Affiliations:** 1 Laboratory of Analytical Chemistry, Department of Chemistry, National and Kapodistrian University of Athens, Panepistimiopolis Zografou, 15771 Athens, Greece; makatsa@chem.uoa.gr (M.K.); npapalou@chem.uoa.gr (N.P.); mtheodwra@gmail.com (T.M.); eirinip97@gmail.com (I.P.); makostak@chem.uoa.gr (M.K.); 2 Laboratory of Food Chemistry, Department of Chemistry, National and Kapodistrian University of Athens, Panepistimiopolis Zografou, 15771 Athens, Greece; harpro@chem.uoa.gr

**Keywords:** fat-soluble vitamins, saponification, enzymatic hydrolysis, extraction, HPLC-DAD, LC-APCI-MS/MS, baby foods, food analysis, estimation of uncertainty

## Abstract

Two liquid chromatographic systems, one coupled to atmospheric pressure chemical ionization and tandem mass spectrometric methods (UHPLC-APCI-MS/MS) and the other a high-performance liquid chromatographic coupled to diode array detector (HPLC-DAD) were used to develop and validate methods for the simultaneous determination of fat-soluble vitamins A, D3 and E in rice cereal baby foods. The chromatographic separation was performed on C18 columns with a mixture of methanol-acetonitrile as mobile phase for all methods. The extraction of fat-soluble vitamins included enzymatic hydrolysis with α-amylase, saponification, extraction with petroleum ether or n-hexane and purification with silica cartridge (only for vitamin D3). Quantification of vitamin D3 and E through UHPLC-APCI-MS/MS was performed by the use of internal standards (IS) D3-d3 and E-d6, respectively, while IS was not used for vitamin A. The methods were optimized and validated in terms of linearity, precision, trueness, limits of detection and quantification. The recoveries were in the range of 85.0–107% for retinol, 92.0–105% for α-tocopherol and 95.2–106% for cholecalciferol and the %RSD (Relative Standard Deviation) values ranged from 6.4% to 15%. The evaluation of the methods was also conducted through the estimation of uncertainties, the application in commercial samples and the participation in a proficiency test

## 1. Introduction

Vitamins are organic compounds that differ in chemical structure, biological activity, and physicochemical properties. Based on their solubility, vitamins are classified into water-soluble (B-complex, and C) and fat-soluble (A, D, E and K) [1]. Vitamins have vital functions especially during periods of increased needs such as pregnancy, growth periods and under the conditions of intensive work. Serious health problems can be caused by their lack or excess. A balanced diet should provide the required amounts of vitamins that are necessary for the human body [2,3,4]. Due to the need for higher vitamin intake, the food industry has started to produce fortified foods with a nutritional label claim to replace all the losses during storage and processing [5].

Fat-soluble vitamins (FSVs) can coexist on the lipid fraction of foods with other lipid constituents (triglycerides, sterols, phospholipids, etc.), a fact that makes their isolation and determination quite complex. FSVs can be found with various chemical forms called vitamers [6,7]. Vitamin A consists of a group of retinoids (retinol, retinal, retinoic acid and retinyl esters) and carotenoids that plays an important role in normal vision, cell growth, normal formation and maintenance of the organs. Vitamin E is a group of eight vitamers, four tocopherols and four tocotrienols composed as α-, β-, γ-, δ-, depending on the number and the position of methyl groups on the chromanol ring [1,8]. The esters of vitamin E were added for food fortification due to their better stability [9]. As they cannot be synthesized in our bodies, they should be sourced through the diet [2]. Vitamin D appears in two major forms; ergocalciferol (D2) and cholecalciferol (D3). It can be synthesized through exposure to UV-B radiation. Cholecalciferol is mostly presented in foods of animal origin such as oil-rich fish and dairy products, while ergocalciferol can be found in some mushrooms, yeasts and fortified foods [10,11].

Due to the complexity of the matrices and the stability of the vitamins, the development and the validation of fast and simple methods is a challenging task. Multiple methods have been developed in various matrices including foods, pharmaceuticals, biological samples and feeds [12]. For the extraction of FSVs from baby foods and infant formulas, various procedures with saponification have been tested [13,14,15]. Liquid chromatography is the most common technique for the quantitative determination of vitamins, coupled to a variety of detectors such as ultraviolet-visible (UV/Vis), diode array detection (DAD), fluorescence (FL), and mass spectrometry (MS^n^). Mass spectrometry is selected because of its high selectivity and sensitivity [16]. Recent advances in liquid chromatography–tandem mass spectrometry (HPLC-MS/MS) have significantly improved the quantification of vitamins at the parts-per-million (mg kg^−1^) and parts-per-billion (μg kg^−1^) levels due to its sensitivity and selectivity, as reported in several papers [17]. HPLC-APCI-MS/MS is a promising technique for the analysis of FSVs as less matrix interferences is observed in APCI source compared to electrospray ionization (ESI) source [18]. It is used increasingly for the analysis of vitamins in various matrices such as infant formulae [19], human plasma [20], vegetables [21], milk [2], and plant foods [22]. In addition, various international (AOAC, CEN, ISO) and national (GB) official methods have been developed in different food matrices [7,23,24,25,26]. These methods present advantages in terms of accuracy but have significant drawbacks. Their experimental procedures are time-consuming and their cost is high due to the large amount of reagents and organic solvents. Thus, there is a need for fast, eco-friendly, fit for purpose methods that can easily be used by companies for daily routine analysis [8].

The aim of this work was to accomplish a comparative study between a simple, fast, eco-friendly, fit-for purpose in-house method and two official Chinese Standard methods for the determination of vitamins A, D3, and E in rice cereal baby foods. To achieve this, two liquid chromatography techniques (HPLC-DAD and UHPLC-APCI-MS/MS) were used in order to compare experimental procedures and evaluate methods through the validation results and the estimation of uncertainties. Multiple methods for the determination of vitamins in milk and cereal-based baby foods have already been published but to the best of our knowledge this is the first comparative study between an in-house method and an official Chinese standard method for the determination of fat-soluble vitamins in rice cereal baby foods including the estimation of the methods’ uncertainty.

## 2. Materials and Methods

### 2.1. Chemicals and Reagents

All the standards (retinol, retinyl acetate, dl-α-tocopherol, α-toco-pheryl acetate and cholecalciferol), the isotope- labeled internal standards of cholecalciferol (6,19,19-d3 solution) and α-tocopherol (phenyl-5,7-dimethyl-d6)) as well as L-ascorbic acid, butylated hydroxyl-toluene (BHT), α-amylase from Aspergillus oryzae and sodium hydroxide were purchased from Sigma Aldrich (Steinheim, Germany). Methanol, ethanol, and acetonitrile HPLC grade, and absolute ethanol and petroleum ether analytical grade, were obtained from Fischer Scientific (Geel, Belgium). Acetonitrile and methanol LC-MS grade were acquired from Merck (Darmstadt, Germany), while anhydrous sodium sulfate was purchased from Honeywell (Offenbach, Germany). Distilled water was provided by a MilliQ purification apparatus (Millipore Direct-Q UV, Bedford, MA, USA). Chromafil Regenerated Cellulose (RC) syringe filters (15 mm diameter, 0.2 mm pore size) were obtained from Macherey-Nagel (Düren, Germany). Solid-phase extraction (SPE) Strata Silica 500 mg/6 mL cartridges (55 m, 70Å) were purchased from Phenomenex (Torrance, CA, USA).

### 2.2. Preparation of Standard Solutions

Stock standard solution of 1000 mg L^−1^ was prepared monthly by weighting 10 mg of each vitamin in a 10 mL volumetric flask, diluted to volume with absolute ethanol. Intermediate standard solutions of 100 mg L^−1^ of retinol, retinol acetate, α-tocopherol, α-toco-pheryl acetate and cholecalciferol were prepared in absolute ethanol. Working standard solutions were prepared daily from the intermediate solutions, in order to construct the calibration curves and the standard addition curves of the analytes. To avoid degradation, the stock solutions were purged under nitrogen steam and stored in amber glass vials at −20 °C for up to one month.

### 2.3. Instrumentation and Chromatographic Conditions

#### 2.3.1. HPLC-DAD

An Agilent HPLC system (1200 infinity series) equipped with an autosampler G1329A, degasser G1379B, column thermostat G1330B, binary pump G1312A, and diode array detector G1315D was used for the determination of vitamin A and E. The chromatographic separation was performed using a C18 column Zorbax Eclipse XDB (5 µm, 250 mm × 4.6 mm) from Agilent (Santa Clara, CA, USA). The column thermostat was set at 35 °C and the mobile phase consisted of (A) methanol (95%) and (B) acetonitrile (5%). The elution program was isocratic, the flow rate was 0.6 mL min^−1^ and the total chromatogram run required 20 min. DAD detector was set to 329 nm for vitamin A and 294 nm for vitamin E. The injection volume was 20 µL. Vitamins A and E were identified through their retention times (3.99 and 9.99 min respectively) and were quantified using standard addition calibration curves. The software used for data treatment was Agilent LC Chemstation Rev. B.01.03-SR2 (204) (Santa Clara, CA, USA, 2004).

#### 2.3.2. UHPLC-APCI-MS/MS

A Thermo TSQ Quantum Access triple quadrupole system, equipped with APCI source, a UHPLC pump (Thermo Accela) and an Accela autosampler was used for the determination of vitamins A, D3 and E. The triple quadrupole mass spectrometer was operated in multiple reaction monitoring (MRM) mode and in positive ionization mode. An ACQUITY UPLC BEH C18 column (1.7 μm, 100 mm × 2.1 mm) from Waters (Milford, MA, USA) equipped with a guard column was used for Methods II and III respectively and thermostated at 30 °C. The mobile phase consisted of (A) methanol (90%) and (B) acetonitrile (10%), using an isocratic elution. The flow rate was 0.5 mL min^−1^ and the run time was 10 min. The injection volume was 10 µL. Instrument control and data acquisition were performed with Xcalibur software, Version 2.3, from Thermo Fisher Scientific (Waltham, MA, USA).

Regarding the MS parameters, multiple reaction monitoring (MRM) was used and two transitions were selected for the identification. The optimum APCI parameters (discharge current, sheath gas, auxiliary gas, vaporizer temperature, capillary temperature), as well as the optimum collision energy (CE) and tube lens value for each vitamin, were obtained by direct infusion of individual standard solutions at a concentration of 1 mg L^−1^ in methanol. Thus, the following MS parameters, discharge current 4.0 μA, sheath gas 35 a.u., auxiliary gas 15 a.u., vaporizer temperature 350 °C and capillary temperature 270 °C, were chosen for analysis. The MRM transitions of this study and the retention time of the vitamins are presented in Table 1. As quantifier ion, the most abundant ion was selected while, as qualifier ion, the second most abundant ion was chosen. The ion ratio (quantifier ion/qualifier ion) of each vitamin constitutes an important identification point and should be within the tolerance limits according to the guidelines of the European Commission Decision 2002/657/EC in order to verify the presence of the analyte in the tested sample [27].

### 2.4. Samples

The samples were commercially obtained from Greek food markets. Baby foods based on rice cereal were analyzed and the method validation was realized in blank baby food samples (free of vitamins). The available forms of fat-soluble vitamins in the commercial samples were retinyl acetate, α-toco-pheryl acetate and cholecalciferol. Vitamin K was not contained in the samples.

### 2.5. Sample Preparation-Extraction of Fat-Soluble Vitamins

**Figure 1:**
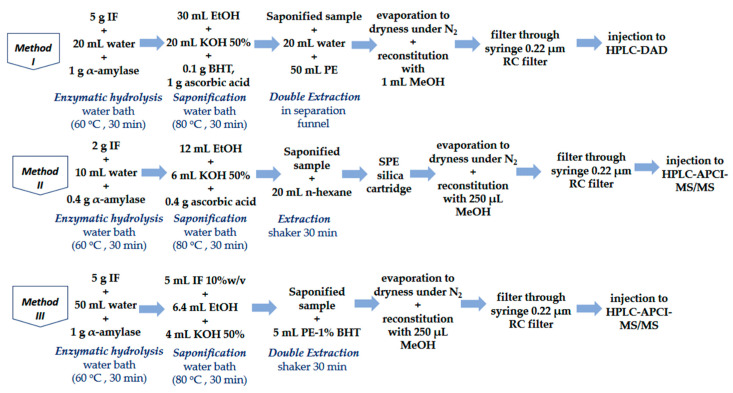
Schematic illustration of the experimental procedures of Methods I-III.

Three methods for the determination of fat-soluble vitamins ADE were optimized and validated: Method I for the determination of vitamins A and E through HPLC-DAD based on the Chinese Standard GB 5009.82 2016 [28], Method II for the determination of vitamin D3 through UHPLC-APCI-MS/MS based on the Chinese Standard GB 5009.82 2016 [28], and Method III for the simultaneous determination of vitamins ADE through UHPLC-APCI-MS/MS based on the method described on our previous work [29]. Briefly, the experimental procedures are illustrated in Figure 1. The principle of all methods was based on the enzymatic hydrolysis, hot saponification and liquid-liquid extraction of the vitamins. During the saponification step, the esters of the vitamins (retinyl acetate and α-toco-pheryl acetate) were converted to their alcohol forms (retinol and α-tocopherol.). All the tubes were covered with aluminum foil and all the procedures were performed under subdued light, to prevent photo degradation of vitamins.

#### 2.5.1. Method I (GB_A&E)

Extraction was carried out according to the procedure described in Standard GB 5009.82 2016 with some modifications. For this purpose, 5 g of homogenized sample was accurately weighed into a 250 mL flat-bottomed flask. Subsequently, 20 mL of warm water was added and the flask was shaken by hand. 1 g α-amylase was added and the sample was placed in a water bath at 60 °C for 30 min. Then, 1 g of ascorbic acid, 0.1 g of BHT, 30 mL of absolute ethanol and 20 mL of 50% aqueous KOH solution were added. The sample was mixed again and placed in a water bath at 80 °C for saponification for 30 min in order to convert the vitamin esters to their alcohol forms. After the saponification step, the flask was immediately placed in an ice bath to cool to room temperature. For the extraction of FSVs, the saponification solution was transferred into a 250 mL separation funnel; 30 mL of water and 50 mL of petroleum ether were added and the mixture was shaken for 5 min. The organic upper phase was transferred into another 250 mL separation funnel and the lower phase was extracted one more time with petroleum ether. The organic layers were combined and washed three times with 100 mL of water. The lower aqueous phase was discarded, and the washed ether layer was filtered through 3 g anhydrous sodium sulfate into a 250 mL round flask. 15 mL of petroleum ether were added to rinse the separation funnel twice. The organic layer was evaporated with the use of a rotary evaporator at 40 °C up to 2 mL. The final extract was transferred to a glass tube and then evaporated to dryness under a nitrogen stream, reconstituted in 1 mL of methanol, filtered through a 0.22 μm RC filter, transferred to a glass vial and injected to the HPLC system.

#### 2.5.2. Method II (GB_D3)

A 2 g portion of each homogenized sample was weighed into a 50 mL polypropylene centrifuge tube. Afterward, spiking of the samples with 60 μg L^−1^ of vitamin D3-d3 internal standard was performed and the samples were left for 30 min. Subsequently, 10 mL of warm water and 0.4 g of α-amylase were added and the sample was mixed for 1 min and placed in a water bath at 60 °C for 30 min. Then, 0.4 g of ascorbic acid, 12 mL of absolute ethanol and 6 mL of 50% aqueous KOH solution were added. The sample was mixed again and placed in a water bath at 80 °C for saponification for 30 min. The cooled saponification solution was centrifuged at 4000 rpm for 5 min and the supernatant was decanted into a new centrifuge tube. After the addition of 20 mL of n-hexane, the sample was mixed thoroughly for 20 min using a mechanical shaker. Thereafter, the sample was centrifuged at 4000 rpm for 3 min and the supernatant was transferred into a new 50 mL centrifuge tube. Then, 25 mL of water were added and the tube was shaken slightly 30 times. The sample was centrifuged at 4000 rpm for 3 min again and the upper organic layer was loaded to an SPE silica cartridge (500 mg/6 mL). The column was activated with 8 mL of ethyl acetate and equilibrated with 8 mL of n-hexane. Then, the sample was passed through the column and the column was rinsed with 6 mL of ethyl acetate:n-hexane solution (5:95) and eluted with 6 mL of ethyl acetate: n-hexane solution in the proportion of 15:85. The eluent was evaporated to dryness at 40 °C under nitrogen steam and reconstituted with 250 μL of methanol. The sample was filtered through a 0.22 μm RC filter, transferred into a glass vial and injected in the UHPLC-APCI-MS/MS system.

#### 2.5.3. Method III (In-House ADE)

5 g of sample were weighed into a 50 mL polypropylene centrifuge tube, spiked with 60 μg L^−1^ of vitamin D3-d3 and 100 μg L^−1^ of vitamin E-d6 and left for 30 min. Afterward, 1 g of α-amylase and 50 mL of warm water (60 °C) were added in order to prepare a 10% *w/v* solution. The sample was stirred until complete homogenization and then the sample was placed in a water bath at 60 °C for 30 min. For the saponification step, 5 mL of the homogenized sample (10% *w/v*), 6.4 mL of ethanol containing 0.2% *w*/*v* ascorbic acid and 4 mL of aqueous KOH 50% *w/v*, were added into a new 50 mL centrifuge tube. The tube was purged with nitrogen for 20 s for the protection of the vitamins from oxygen degradation and shaken for a few seconds. Subsequently, the sample was placed in a water bath at 80 °C for 30 min and then cooled in an ice bath for a few minutes. The saponified sample was centrifuged at 4000 rpm for 5 min and then transferred to a new 50 mL centrifuge tube. The extraction of FSVs was conducted with the addition of 5 mL of petroleum ether containing 1% *w/v* BHT. The sample was mixed thoroughly for 20 min using a mechanical shaker and was allowed to rest for 2–3 min, as the two phases were separated completely. The upper organic phase was collected in a glass tube. The above-mentioned extraction procedure was repeated and the organic phases were again collected to the same glass tube and evaporated under gentle nitrogen steam to dryness and then were reconstituted with 250 μL of methanol. The extract was filtered through a 0.22 μm RC filter, transferred to a glass vial and injected in the UHPLC-APCI-MS/MS system.

### 2.6. Method Validation

The three methods were validated in terms of linearity, trueness (recovery), precision (repeatability, reproducibility), methods limit of detection (LOD) and limit of quantification (LOQ) according to Eurachem Method Validation Guide [30]. All the validation experiments were carried out using the standard addition method by spiking blank baby food samples (free of vitamins) with the proper amounts of vitamin’s standards. The use of isotope-labeled internal standards was only feasible for vitamin D3 (Method II and III) and vitamin E (Method III) through UHPLC-MS/MS analysis because their detection and their separation from the analyte were not possible through HPLC-DAD. Thus, for vitamins D3 (Method II and III) and E (Method III), which their internal standards were available, the ratio of the peak area of the analyte to the peak area of its corresponding internal standard was used for quantification purposes and the correction of the results. In case of vitamins A (Method I and III) and E (Method I), where internal standards were not applied, the peak areas of the analytes were used for the quantification of these vitamins.

Linearity was assessed by constructing standard calibration curves and standard addition curves for each vitamin in eight different concentration levels and expressed as coefficient of determination (*r*^2^). In the standard calibration curves, the concentrations ranged from 0.6 to 50 mg L^−1^ for retinol, from 7.00 to 500 mg L^−1^ for α-tocopherol and from 0.008–0.480 mg L^−1^ for cholecalciferol. As for the standard addition curves, vitamin concentrations were in the range of 0.300–15.0 mg kg^−1^ for retinol, 3.50–150 mg kg^−1^ for α-tocopherol and 0.008–0.240 mg kg^−1^ for cholecalciferol. For Method II and III, matrix-matched standards were also analyzed to investigate the matrix effect. Precision was evaluated through repeatability and intermediate precision experiments and expressed as the relative standard deviation (% RSD). Trueness was calculated through the recoveries of the spiked samples. The samples were spiked at three different fortification levels for each vitamin (A: 1.75, 3.50 and 7.00 mg kg^−1^, D3: 40.0, 80.0 and 160 μg kg^−1^ and E: 17.5, 35.0 and 75.0 mg kg^−1^) in six replicates on two different laboratory days under the same conditions. The LOD and LOQ of each method and for each analyte were determined by spiking the analytes at the lowest concentration level that can be detectable in the instrument in ten replicates.

### 2.7. Assessment of Methods

#### 2.7.1. Measurement Uncertainty

To evaluate the methods, the estimation of uncertainty was conducted. Validation data were used according to Eurachem Guide [31]. The uncertainty was calculated for each method and for each vitamin at three concentration levels. In the combined uncertainty (u_combined_), the uncertainties associated with sample mass (u_m_), volume (u_v_), recovery (u_bias_), repeatability (u_random_), and calibration curve (u_calibration_) were taken into consideration. Finally, the expanded combined uncertainty (U) was calculated by multiplying by a coverage factor k that was set to 2 for a confidence level of 95% [31,32,33].

#### 2.7.2. External Quality Control and Real Samples

The participation of our laboratory in a proficiency test was considered an important part of the current study to confirm the accuracy of the methods. Due to the difficulty in finding a proficiency test similar to samples used in this study, a baby milk from BIPEA (11-2520) was chosen, as the closest matrix to the analyzed samples.

In addition, the applicability of the methods was demonstrated by analyzing rice cereal baby food samples from market. Three replicates of four real samples were analyzed in each method in order to compare the results with the labeling values.

## 3. Results and Discussion

### 3.1. Method Optimization

#### 3.1.1. Method I (GB_A&E)

The experimental procedure for Method I was described in Section 2.5.1. Some modifications were realized only in the chromatographic conditions of the proposed method [28]. As for the chromatographic conditions, a C18 column was chosen instead of the proposed C30 column. In the official method, the determination of (α, β, γ, δ-tocopherols) was conducted, while in our samples only α- tocopherol needed to be determined as it was the only tocopherol contained in the analyzed samples. The constitution of the mobile phase was optimized and better chromatographic peaks can be obtained with MeOH:ACN (95:5) than 100% methanol. Finally, the quantification of the samples was conducted by standard addition method due to the matrix effect, as well as in Methods II and III.

#### 3.1.2. Method II (GB_D3)

**Figure 2:**
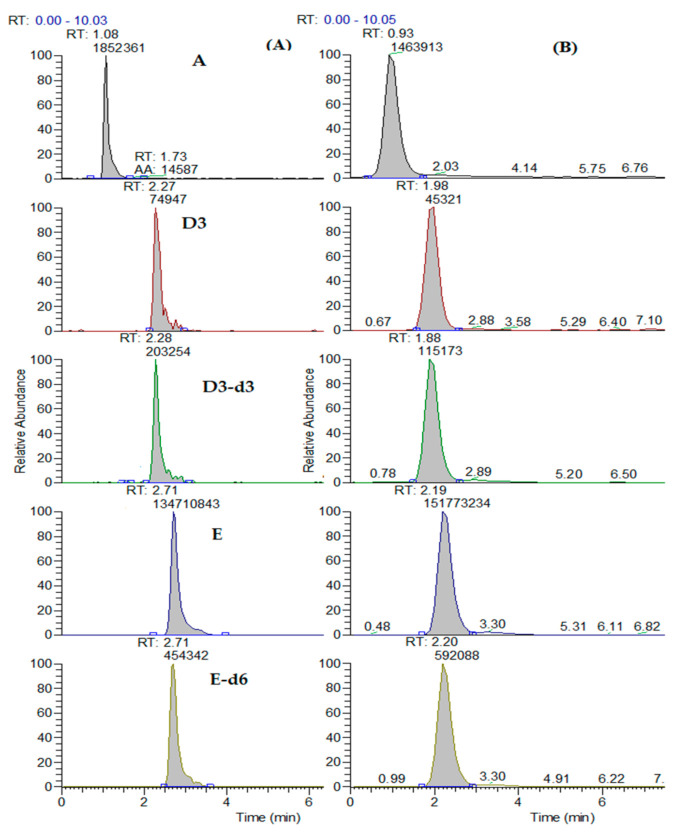
Comparative chromatogram of a mix standard solution through LC-APCI-MS/MS (atmospheric pressure chemical ionization and tandem mass spectrometric methods) with columns (**A**) Atlantis T3 and (**B**) ACQUITY UPLC BEH C18. RT: retention time.

The experimental protocol was described in Section 2.5.2. The only parameter optimized in the experimental procedure was the constitution and the volume of the reconstitution solution in order to be compatible with the mobile phase and to preconcentrate the analyte (eight times). Regarding the UHPLC-MS/MS parameters, APCI was chosen as the best source for the determination of vitamin D3 because the sensitivity of the ionization of the analyte with the ESI was not satisfactory. In order to find the optimum mobile phase, two were tested; the proposed one with 5 mM ammonium acetate buffers and the second with a mixture of MeOH (90%) and ACN (10%). The second one was chosen, as sensitivity and peak symmetry of vitamin D3 were improved significantly. Finally, two columns were tested; an Atlantis T3 (3μm, 100 mm × 4.6 mm) and an ACQUITY UPLC BEH C18 (1.7 μm, 100 mm × 2.1 mm) No difference was observed in the sensitivity of the analytes for the two columns, whereas a significant difference was noticed regarding the peak shape and the symmetry of the chromatographic peaks, as shown in Figure 2. Therefore, the ACQUITY UPLC BEH C18 column was chosen because the chromatography was improved due to the smaller particle size (1.7 μm) and inner diameter (2.1 mm), as the two columns had the same length (100 mm).

The goal of this method was to develop a simple, fast and fit-for-purpose method for the simultaneous determination of ADE. According to the literature, most of the methods for the determination of FSVs are time-consuming and require large amounts of reagent, including the described Methods I and II [7,24,26]. This method protocol was based on a previous study in baby foods based on milk and cereals [29]. The experimental procedure was modified to achieve preconcentration of the sample and to detect not only vitamins A and E but also vitamin D3, whose concentration in samples is measured in μg kg^−1^. For this reason, different masses of the samples weighted, varying from 2 to 5 g of sample solution (10% *w/v*), were tested. 5 g were chosen as the optimum mass and 2 times preconcentration of the sample was achieved. For the determination of ADE the chromatographic conditions of Method II were chosen. Additionally, in this method the flow rate was investigated from 0.4 up to 0.6 mL min^−1^. We concluded that, when the flow rate was set at 0.5 mL min^−1^, the best sensitivity of all the analytes was achieved. It is noteworthy to mention that, for the selected flow rate, the intensity of the chromatographic peaks was doubled.

### 3.2. Method Validation

#### 3.2.1. Linearity

All the methods were validated and satisfactory results were obtained according to the AOAC Guidelines for Standard Method Performance Requirements [34]. The evaluation of linearity was conducted by least-squares linear regression analysis and linear regression values (*r*^2^ > 0.990) for the standard calibration curves, as well as for the standard addition curves. The quantification of the analytes was carried out using standard addition curves in all the above-mentioned methods to compensate for matrix effect.

Blank baby food samples were fortified in eight different concentration levels with the analytes of our interest and the internal standards of vitamins D3 and E for the construction of standard addition curves. Afterwards, eight blank samples were extracted, and these extracts were used for the construction of matrix-matched calibration curve. The concentration levels for external standard, matrix matched and standard addition calibration curves were the same in all cases and selected by taking into consideration the preconcentration of the samples (5 times for Method I, 8 times for Method II and 2 times for Method III).

The linearity results and the concentration range of each analyte are presented in Table 2, Table 3 and Table 4. Based on these results, a good linearity was proven as all the linear regression values are satisfactory in all the methods. For Methods II and III matrix-matched curves to evaluate the matrix effect through UHPLC-MS/MS analysis were also selected.

#### 3.2.2. Precision-Trueness

The accuracy of the methods was investigated through the evaluation of repeatability and intermediate precision. The recovery and the %RSD of each analyte were examined in three different fortification levels in six replicates in two different laboratory days. The results of the intermediate precision of this study are presented in Table 5 and Table 6.

It was observed that for all the methods and all the analytes, acceptable levels of precision (%RSD_R_ < 16%) and trueness (recoveries 80%–110%) were achieved according to AOAC Guidelines [34]. More specifically, the % RSD_R_ values were lower than 15% and the recoveries ranged within 85%–107% for all the methods. Comparing the results of the methods for vitamin A, it was noticed that %RSD_R_ of Method III was slightly higher (~12.8%) comparing to %RSD_R_ of Method I (~10.3%) but the recoveries are almost the same. The only differentiation in the recoveries was observed for the low level, where the recovery of Method I was significantly lower (~85%) than the one obtained from Method III (~103%). This might have occurred due to the matrix interferences as retinol eluted at the beginning of the chromatogram. For vitamin E, %RSDs and the recoveries were similar for all the fortification levels. In contrast, the results of vitamin D3 varied between the two methods as the experimental procedures presented significant differences. As shown in Table 5, %RSDs of Method II were lower (~10.9%) than Method III (~12.9%) due to the purification step with SPE after the extraction.

#### 3.2.3. LODs and LOQs

LODs and LOQs of the methods were estimated by analyzing ten blank samples. In particular, LODs were determined by 3.3 times the SD of the peak area of the vitamin divided to the slope of its standard addition calibration curve. LOQs were calculated by multiplying the LODs three times. The instrumental LODs and LOQs were determined in the same way but from the standard calibration curves. In cases in which the isotope-labeled internal standards of vitamins D3 and E were used, the ratio of the peak area of the analyte to the peak area of its corresponding internal standard was chosen for the calculation of LODs and LOQs of these vitamins. The results are summarized in Table 7 and Table 8. In all cases, the methods’ LOD and LOQ were satisfactory as they were significantly lower than the expected level. Comparing the method’s LODs of vitamin A between the two liquid chromatography techniques, it was noticed that they were similar (0.36 mg kg^−1^ for Method I and 0.30 mg kg^−1^ for Method III), possibly due to the strong matrix effect in LC-MS/MS. However, the instrumental and method LODs of vitamin E were significantly lower in LC-MS/MS than in HPLC-DAD as was expected, due to the higher sensitivity of the analyte. The LODs of vitamin D3 comparing Methods II and III were similar as the same chromatographic conditions and detection technique were used for its determination.

#### 3.2.4. Matrix Effect

In LC-MS/MS measurements, the matrix effect is a parameter that has to be studied in order to investigate the effect of the matrices on the detection, especially when analyzing complex food samples like baby foods and infant formulas. The matrix effect can have an impact on the ionization efficiency, and suppression or enhancement of the signal can be noticed. As the result, standard addition or matrix-matched curves were used for the quantification of the samples. Thus, the matrix factor (*MF*) and the matrix effect (*ME*) were calculated comparing the slopes from matrix-matched calibration curves and standard calibration curves according to the following Equations (1) and (2):

(1) $$MF\;=\frac{Slope\;“matrix-matched\;curve”}{Slope\;“standard\;curve”}$$

(2) $$\%ME=\left(MF-1\right)\times 100$$

Positive *ME* value signifies signal enhancement, while a negative *ME* value indicates signal suppression. When the %*ME* is bigger than ± 20%, then the matrix effect is strong [35]. Due to the complexity of rice cereal, a strong matrix effect was expected for all the analytes. However, the use of APCI as an ionization source less susceptible to matrix effect contributes to the decrease in this phenomenon.

As shown in Table 9, a strong matrix effect (~47%) was only presented in vitamin A. This probably occurred as retinol is eluted at the beginning of the chromatogram (0.84 min), and for its quantification an internal standard was not used, in contrast to vitamins D3 and E. Comparing the ME of vitamin D3 in Methods II and III, the same percentage of matrix effect was notices, although Method II reveals signal enhancement while Method III reveals signal suppression. As a result of the presence of this strong matrix-effect, the quantification of the samples was conducted by standard addition curves instead of external standard calibration curves.

### 3.3. Assessment of Methods

#### 3.3.1. Measurement Uncertainty

The uncertainty of the measurement was estimated according to the Eurachem Guide in order to compare the validation results and evaluate all the methods [31]. In Table 10 the uncertainties are presented, with an impact on the measurement of the combined uncertainty of each concentration level for each vitamin, as well as the expanded combined uncertainty (U).

**Figure 3:**
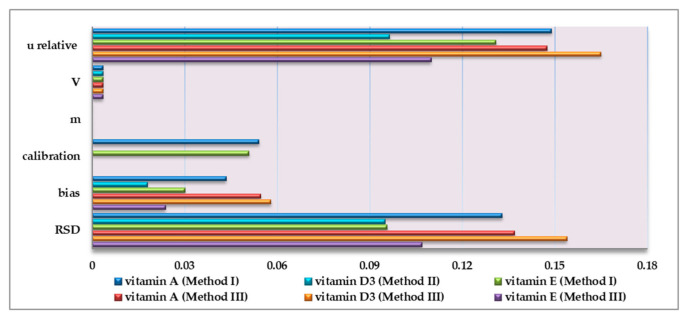
Uncertainty contribution (u relative) in the medium fortification level for all the vitamins taking into consideration the uncertainties of volume (V), sample mass (m), calibration curve (calibration), recovery (bias) and repeatability (RSD).

As shown in Figure 3, u_bias_ and u_random_ made the higher contribution to the combined uncertainty in all the methods. In the UHPLC-APCI-MS/MS methods u_calibration_ was not calculated, as the standard addition curves were prepared on each experimental day and its uncertainty was included with u_random_.

When comparing the combined uncertainty of the methods, it was observed that uncertainty for vitamin A and E in Method III was slightly lower than in Method I. In contrast, the uncertainty of vitamin D3 was slightly lower in Method II than in Method III. This fact possibly occurred due to the presence of the SPE step that helped the purification and the preconcentration of the vitamin.

Briefly, there is no significant difference between the uncertainties of the official (Method I & II) and the in-house method (Method III). As a result, the in-house method can be considered equivalent to the two official methods.

#### 3.3.2. External Quality Control and Real Samples

The participation in a proficiency test confirmed the accuracy of the methods. The results were within the z-score limits as presented in Table 11. The concentrations of vitamin A (4.69 and 4.53 mg kg^−1^) and vitamin D3 (0.0900 and 0.0916 mg kg^−1^) were very close when comparing the two methods, while differences were observed between the methods for vitamin E, as the result obtained with Method III (128 mg kg^−1^) was closer to the assigned value (154 mg kg^−1^).

Finally, the applicability of the methods was tested by analyzing four different types of commercial baby foods in three replicates. According to the European Commission Guidance, tolerances of nutrient values are declared on foods label. In particular, the tolerances for vitamins ranged between +50% and −35% of the declared value, including the uncertainty of the measurement [36]. As shown in Table 12, the measured concentrations of the vitamins were similar and the standard deviation of the samples was slightly higher in Method III than those in Methods I and II. In all cases the results were acceptable and within tolerance limits and the methods are suitable for routine analysis.

**Figure 4:**
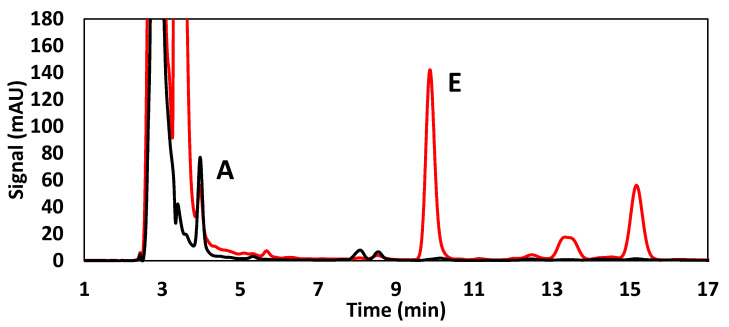
Chromatographic separation of vitamin A and E through high-performance liquid chromatographic coupled to diode array detector (HPLC-DAD) with Method I in real baby food sample.

**Figure 5:**
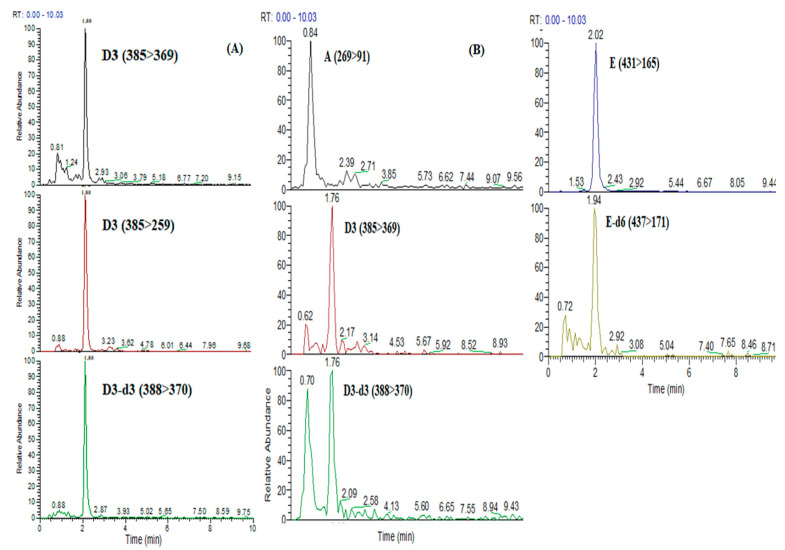
Chromatographic separation of (**A**) vitamin D3 with Method II and (**B**) vitamins A, D3, D3-d3 (IS), E and E-d6 (IS) with Method III through LC-APCI-MS/MS in real baby food sample.

In Figure 4 and Figure 5, the typical chromatograms of the determination of FSVs in real samples with each method were illustrated.

## 4. Conclusions

In this current study, three methods for the determination of fat-soluble vitamins in rice cereal baby foods have been optimized and fully validated: one HPLC-DAD method for the determination of vitamins A and E, one UHPLC-APCI-MS/MS method for the determination of vitamin D3 and one UHPLC-APCI-MS/MS for the simultaneous determination of ADE. The benefit of the methods was the hot saponification step that helped the extraction of fat-soluble vitamins from complex matrices such as the baby foods and the infant formulas. All the methods demonstrated acceptable performance characteristics confirming their suitability. Methods I and II were based on official Chinese methods with some modifications but they were complicated, time-consuming and expensive because of the large amount of reagents. In contrast, Method III (for the simultaneous determination of ADE) was faster (2-h experimental procedure), more eco-friendly and a cheaper method for routine analysis. However, a strong matrix effect for vitamin A was observed comparing to that noticed for vitamins D3 and E. To overcome this difficulty, the quantification of the vitamins was conducted by the standard addition curve. Moreover, the use of an internal standard of vitamin A could be an alternative for the amelioration of the results; however, the purchase of this internal standard was not affordable [3,22].

Additionally, all the methods were successfully applied in a proficiency test sample of baby milk and the z-scores achieved for all the analytes were below 2, indicating satisfactory accuracy of the proposed methods. These results showed that the methods could also be applied in other types of baby foods such as milk and cereal-based baby foods that are less complicated matrices. Finally, the assessment of the methods was conducted through the estimation of uncertainties. The results showed that the in-house method presented better or similar uncertainty to the official Chinese methods and can be considered reliable for routine analysis for the determination of FSVs in baby foods.

Compared to previous studies, the in-house method was simple, fast and economic as there was no need for a SPE step, large quantities of organic reagents and overnight saponification. As far as the results are concerned, our method demonstrated satisfactory accuracy and precision as similar results when compared to other published studies, but higher detection limits. This was expected due to the complexity of the examined matrix compared to infant formulas and milk products [3,19,22,37,38,39].

## Figures and Tables

**Table 1:** Multiple Reaction Monitoring (MRM) transitions of the studied analytes.

Compound	Parent Ion (m/z)	Quantifier Ion (m/z)	CE (eV)	Qualifier Ion (m/z)	CE (eV)	Tube Lens	Retention Time (min)
Retinol	269.2	91.4	33	105.2	11	52	0.85
Retinyl acetate	269.2	91.4	33	105.2	11	52	1
Cholecalciferol	385.2	259.1	13	367.2	11	53	1.8
Cholecalciferol-d3	388.4	370	11	259.1	11	68	1.8
α-Tocopherol	431.1	165.2	20	137.2	35	75	2
α-Toco-pheryl acetate	473.4	207.1	17	165.2	21	97	2.5
α-Tocopherol-d6	437.4	171.1	22	143.1	35	65	2

CE: collision energy.

**Table 2:** Standard calibration curves and correlation factors for each analyte.

Compound	Method	Concentration Range (mg L^−1^)	Calibration Curve	Coefficient of Determination (*r*^2^)
Retinol	I	0.88–50.0	y = (90.1 ± 1.2) x + (35.3 ± 26.8)	0.9996
	III	0.600–30.0	y = (326.9 ± 6.6) × 10^3^ x − (17.1 ± 9.2) × 10^4^	0.997
α-Tocopherol	I	9.25–500	y = (0.11 ± 8.02) × 10^2^ x − (6.4 ± 18.2)	0.9996
	III	7.00–300	y = (4.73 ± 0.11) x − (25.2 ± 14.8)	0.996
Cholecalciferol	II	0.005–2.56	y = (76 ± 1.11) × 10^−4^ x + (16.25 ± 1.15) × 10^−2^	0.9998
	III	0.008–0.480	y = (69.9 ± 3.4) × 10^−4^ x + (3.1 ± 77.7) × 10^−3^	0.998

**Table 3:** Standard addition calibration curves and correlation factors for each analyte.

Compound	Method	Concentration Range (mg kg^−1^)	Calibration Curve	Coefficient of Determination (r^2^)
Retinol	I	0.750–10.0	y = (360.0 ± 10.6) x + (88.7 ± 58.1)	0.995
	III	0.300–15.0	y = (47.9 ± 1.8) × 10^4^ x + (31.7 ± 12.2) × 10^4^	0.99
α-Tocopherol	I	9.00–100	y = (36.3 ± 1.0) x + (80.7 ± 81.6)	0.995
	III	3.50–150	y = (9.05 ± 0.23) x − (51.9 ± 15.7)	0.995
Cholecalciferol	II	0.008–0.160	y = (55.2 ± 2.4) × 10^−3^ x + (46.4 ± 31.1) × 10^−2^	0.992
	III	0.008–0.240	y = (10.9 ± 6.6) × 10^−3^ x + (57.1 ± 76.1) × 10^−2^	0.998

**Table 4:** Matrix-matched curves and correlation factors for each analyte.

Compound	Method	Concentration Range (mg L^−1^)	Calibration Curve	Coefficient of Determination (r^2^)
Retinol	III	0.600–30.0	y = (145.7 ± 3.8) × 10^3^ x + (44.8 ± 2.6) × 10^4^	0.995
α-Tocopherol	III	7.00–300	y = (4.81 ± 0.15) x − (44.1 ± 21.6)	0.992
Cholecalciferol	II	0.008–0.160	y = (62.8 ± 3.1) × 10^−3^ x + (0.355 ± 0.406)	0.99
	III	0.008–0.480	y = (59.5 ± 3.3) × 10^−4^ x + (16.8 ± 7.5) × 10^−2^	0.98

**Table 5:** Comparative results of recoveries and precision for Vitamins A and E.

Compound	Fortification Level	Method I			Method III		
		Mean Concentration (mg kg^−1^) (*n* = 12)	Mean Recovery (%)	% RSD_R_	Mean Concentration (mg kg^−1^) (*n* = 12)	Mean Recovery (%)	% RSD_R_
Retinol	low	1.49	85.0	7.3	1.80	103	13.2
	medium	3.69	105	13.3	3.43	98.1	13.7
	high	7.48	107	10.4	7.42	106	11.4
α-Tocopherol	low	16.2	92.5	12.7	17.8	102	13.6
	medium	35.7	102	9.57	36.4	104	10.7
	high	77.4	103	10.3	78.9	105	6.4

RSD: Relative Standard Deviation.

**Table 6:** Comparative results of recoveries and precision for Vitamin D3.

Compound	Fortification level	Method II			Method III		
		Mean Concentration (μg kg^−1^) (*n* = 12)	Mean Recovery (%)	% RSD_R_	Mean Concentration (μg kg^−1^) (*n* = 12)	Mean Recovery (%)	% RSD_R_
Cholecalciferol	low	42.1	105	10.3	38.2	95.5	12.5
	medium	84.6	106	9.5	76.2	95.2	14.4
	high	154	96.4	13	158	98.5	10.9

**Table 7:** Instrumental limit of detection (LOD) and limit of quantitation (LOQ) of each vitamin.

Method	Retinol		α-Tocopherol		Cholecalciferol	
	LOD (mg L^−1^)	LOQ (mg L^−1^)	LOD (mg L^−1^)	LOQ (mg L^−1^)	LOD (μg L^−1^)	LOQ (μg L^−1^)
I	0.98	2.9	5.4	16	-	-
II	-	-	-	-	5	15
III	0.43	1.3	1.9	5.7	5	15

**Table 8:** Methods - limit of detection (LOD) and limit of quantitation (LOQ) of each vitamin.

Method	Retinol		α-Tocopherol		Cholecalciferol	
	LOD (mg kg^−1^)	LOQ (mg kg^−1^)	LOD (mg kg^−1^)	LOQ (mg kg^−1^)	LOD (μg kg^−1^)	LOQ (μg kg^−1^)
I	0.36	1.2	0.74	2.4	-	-
II	-	-	-	-	7.7	23
III	0.3	0.91	0.31	0.92	9.2	28

**Table 9:** Matrix factor and matrix effect for each analyte in LC-MS/MS analysis.

Compound	Method	MF	%ME
Retinol	III	0.5	−47
α-Tocopherol	III	1	1.7
Cholecalciferol	II	1.1	14
	III	0.8	−15

**Table 10:** Estimation of the uncertainty of each analyte.

	Method I			Method I			Method II		
Parameter	Retinol (mg kg^−1^)			α-Tocopherol (mg kg^−1^)			Cholecalciferol (μg kg^−1^)		
u_random (RSD)_, %	0.073	0.133	0.104	0.127	0.0957	0.103	0.103	0.095	0.095
u_bias_, %	0.0376	0.0435	0.0462	0.0272	0.0301	0.0283	0.0419	0.018	0.018
u_calibration_, %	0.147	0.0542	0.0284	0.12	0.0509	0.0225	-	-	-
u_m_, % (10^−7^)	5.5	5.5	5.5	5.5	5.5	5.5	5.5	5.5	5.5
u_V_, %	0.0036	0.0036	0.0036	0.0036	0.0036	0.0036	0.0036	0.0036	0.0036
u_relative_, %	0.16	0.15	0.12	0.18	0.13	0.11	0.11	0.1	0.1
u_relative_ (k = 2), %	0.33	0.3	0.24	0.35	0.26	0.23	0.22	0.19	0.19
Mean concentration	1.49	3.7	7.5	16.2	35.7	77	42	85	154
U (k = 2), mg kg^−1^ & μg kg^−1^	0.48	1.1	1.8	5.7	9.4	18	9.4	16	22
	**Method III**								
**Parameter**	**Retinol** (**mg kg^−1^**)			**a-Tocopherol** (**mg kg^−1^**)			**Cholecalciferol** (**μg kg^−1^**)		
u_random (RSD)_, %	0.132	0.137	0.114	0.13	0.107	0.0639	0.125	0.154	0.109
u_bias_, %	0.0667	0.0547	0.0263	0.052	0.0238	0.0188	0.061	0.058	0.0385
u_calibration_, %	-	-	-	-	-	-	-	-	-
u_m_,% (10^−7^)	5.5	5.5	5.5	5.5	5.5	5.5	5.5	5.5	5.5
u_V_, %	0.0036	0.0036	0.0036	0.0036	0.0036	0.0036	0.0036	0.0036	0.0036
u_relative_, %	0.15	0.15	0.12	0.14	0.11	0.07	0.14	0.17	0.12
u_relative_ (k = 2), %	0.3	0.3	0.23	0.28	0.22	0.13	0.28	0.33	0.23
Mean concentration	1.8	3.4	7.4	17.8	36.4	79	38	76	158
U (k = 2), mg kg^−1^ & μg kg^−1^	0.53	1	1.7	5	8	11	11	25	36

**Table 11:** Results of the determination of vitamins in a baby milk proficiency test.

Compound	Assigned Value (mg kg^−1^)	Concentration (z-Score)		
		(mg kg ^−1^)		
		Method I	Method II	Method III
Retinol	4.26	4.69 (0.67)	-	4.53 (0.42)
α-Tocopherol	154	107 (−1.78)	-	128 (−0.98)
Cholecalciferol	0.118	-	0.0900(−1.60)	0.0916 (−1.51)

**Table 12:** Quantitative results of the determination of vitamins in commercial samples.

	**Rice Cereal**			**Farine Lactée**		
**Method**	**Label Value**	**Tolerance limits**	**Concentration (*n* = 3)**	**Label Value**	**Tolerance limits**	**Concentration (*n* = 3)**
I	3.50	2.45-5.25	2.95 ± 0.15	3.90	2.73-5.85	4.12 ± 0.23
III			3.01 ± 0.45			4.23 ± 0.40
I	35.0	24.5-52.5	42.4 ± 2.0	34.00	23.8-51.0	40.9 ± 2.6
III			43.6 ± 3.1			39.8 ± 4.0
II	70.0	50.0-110	73.4 ± 4.3	68.00	47.6-102	69.8 ± 3.1
III			84.0 ± 3.5			64.4 ± 5.0
	**Biscuit Cream**			**Fruit cream with 3 fruits**		
**Method**	**Label Value**	**Tolerance limits**	**Concentration (*n* = 3)**	**Label Value**	**Tolerance limits**	**Concentration (*n* = 3)**
I	4.15	2.91-6.23	4.65 ± 0.19	3.40	2.38-5.10	4.09 ± 0.24
III			4.82 ± 0.29			4.27 ± 0.0.35
I	30.0	21.0-45.0	36.7 ± 3.2	37.0	25.9-55.5	40.7 ± 2.5
III			35.8 ± 3.1			40.4 ± 2.9
II	69.0	48.3-104	69.8 ± 3.6	75.00	52.5-113	79.0 ±4.0
III			64.4 ± 5.0			73.2 ± 4.4

## Data Availability

Data sharing not applicable.
